# Transcription factor roles in the local adaptation to temperature in the Andean Spiny Toad *Rhinella spinulosa*

**DOI:** 10.1038/s41598-024-66127-5

**Published:** 2024-07-02

**Authors:** Fernando Hinostroza, Ingrid Araya-Duran, Alejandro Piñeiro, Isabel Lobos, Luis Pastenes

**Affiliations:** 1 Centro de Investigación de Estudios Avanzados del Maule (CIEAM), Vicerrectoría de Investigación y Postgrado, Universidad Católica del Maule, Talca, Chile; 2 Centro de Investigación en Neuropsicología y Neurociencias Cognitivas, Facultad de Ciencias de la Salud, Universidad Católica del Maule, Talca, Chile; 3 Escuela de Química y Farmacia, Departamento de Medicina Traslacional, Facultad de Medicina, Universidad Católica del Maule, Talca, Chile; 4 Centro Para la Investigación Traslacional en Neurofarmacología, Universidad de Valparaíso, Valparaíso, Chile; 5 Center for Bioinformatics and Integrative Biology, Facultad de Ciencias de la Vida, Universidad Andrés Bello, Santiago, Chile; 6 Laboratorio de Genética y Microevolución, Departamento de Biología y Química, Facultad de Ciencias Básicas, Universidad Católica del Maule, Talca, Chile

**Keywords:** Anuran, DNA-binding protein, Ectotherms, Gene expression, Geothermal streams, Metamorphosis, Transcriptome, Evolution, Genetics, Zoology

## Abstract

Environmental temperature strongly influences the adaptation dynamics of amphibians, whose limited regulation capabilities render them susceptible to thermal oscillations. A central element of the adaptive strategies is the transcription factors (TFs), which act as master regulators that orchestrate stress responses, enabling species to navigate the fluctuations of their environment skillfully. Our study delves into the intricate relationship between TF expression and thermal adaptation mechanisms in the *Rhinella spinulosa* populations. We sought to elucidate the dynamic modulations of TF expression in prometamorphic and metamorphic tadpoles that inhabit two thermally contrasting environments (Catarpe and El Tatio Geyser, Chile) and which were exposed to two thermal treatments (25 °C vs. 20 °C). Our findings unravel an intriguing dichotomy in response strategies between these populations. First, results evidence the expression of 1374 transcription factors. Regarding the temperature shift, the Catarpe tadpoles show a multifaceted approach by up-regulating crucial TFs, including fosB, atf7, and the androgen receptor. These dynamic regulatory responses likely underpin the population’s ability to navigate thermal fluctuations effectively. In stark contrast, the El Tatio tadpoles exhibit a more targeted response, primarily up-regulating foxc1. This differential expression suggests a distinct focus on specific TFs to mitigate the effects of temperature variations. Our study contributes to understanding the molecular mechanisms governing thermal adaptation responses and highlights the resilience and adaptability of amphibians in the face of ever-changing environmental conditions.

## Introduction

Understanding the mechanisms underlying local adaptation to specific environmental factors is fundamental in evolutionary biology^1^. Studies of local adaptation offer invaluable insights into the influence of environmental factors on organism genetic responses, with temperature being a critical factor, especially for ectothermic vertebrates^2^. Ectotherms have limited thermal regulation capabilities, making them highly susceptible to environmental fluctuations^3^. Temperature directly impacts amphibian local adaptation processes^4^. Transcription factors (TFs) orchestrate gene expression in response to internal and external cues, facilitating species' survival and adaptation. As the diversity and adaptation of species hinge on TF activities, comprehending their roles in thermal adaptation is pivotal^5,6^.

*Rhinella spinulosa* Wiegmann, 1834, is a diploid bufonid anuran^7^ that has a wide geographic distribution, ranging from the Peruvian-Bolivian Altiplano to the eastern and western slopes of the Andes in Chile and Argentina (IUCN SSC Amphibian Specialist Group, 2020). In Chile, this species is found from 17°30′ to 41°30′ S latitude and 1400 to 4580 m altitude^8^. Méndez and Correa-Solis^9^ characterized a population of *R. spinulosa* that inhabits the geothermic streams of El Tatio, Chile, an aquatic environment marked by constant temperatures (25 ± 1 °C). This habitat contrasts with the streams of the Catarpe valley, Chile, which experiences oscillating daily temperatures^8^. Interestingly, the El Tatio population cannot adapt to colder temperature shifts, exhibiting survival rates plummeting below 10% and growth rates diminishing to approximately 0.1 mm/day when individuals are exposed to 20 °C^9^. A previous RNA-Seq analysis in *R. spinulosa* tadpoles from El Tatio and Catarpe, which were exposed to temperatures of 25 °C and 20 °C, revealed significant differences in gene expression in response to thermal stress^8^. Despite their pivotal role in gene regulation and species adaptation, the TFs of *R. spinulos*a remain unexplored. Therefore, ascertaining the census of these TFs and elucidating their connection to local thermal adaptation is essential.

The wide distribution of *R. spinulosa* in heterogeneous thermal environments offers a unique opportunity to unravel the complexities of thermal adaptation under specific conditions. Noteworthy, information about molecular mechanisms underlying local thermal adaptation in native populations is limited. Furthermore, studies about the role of TFs in adaptation processes are scarce. Therefore, our study endeavors to reduce the knowledge gap concerning the TFs of *R. spinulosa*, which are vital components in deciphering the mechanisms behind species adaptation. Finally, we seek to establish a connection between these TFs and the local thermal adaptations that this anuran exhibits.

## Results

### Rhinella spinulosa TF census

**Figure 1:**
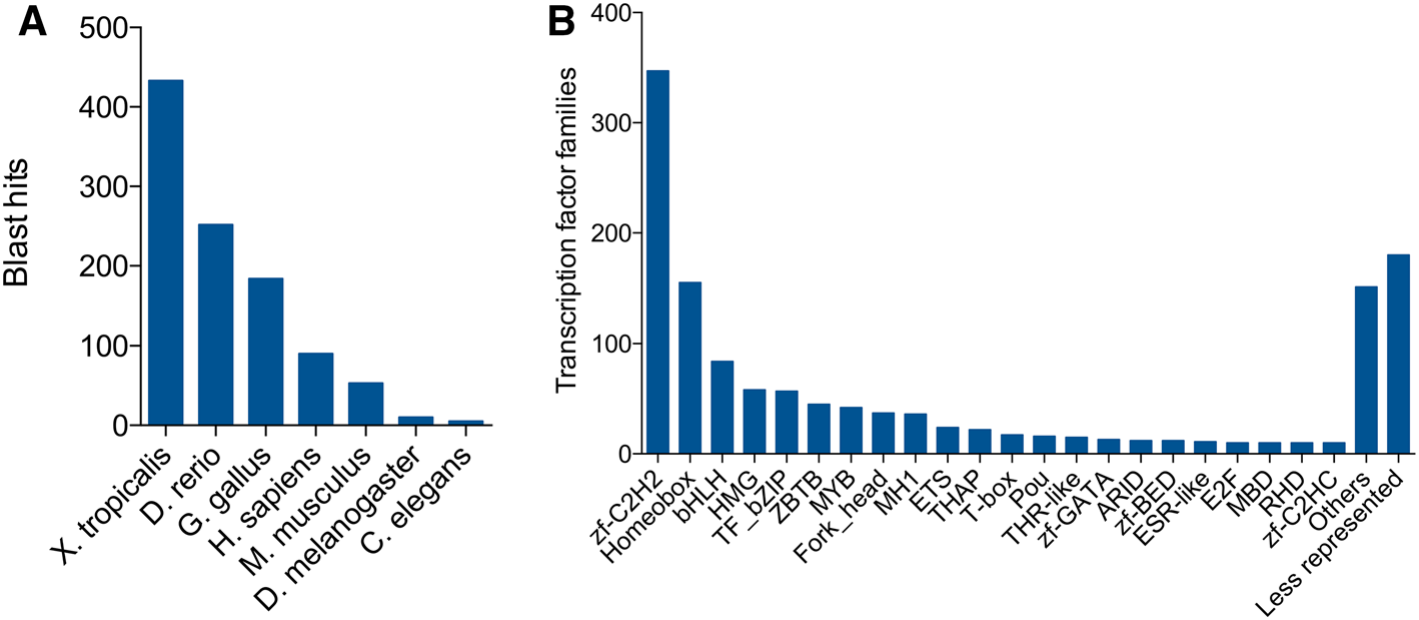
Transcription factor census. (**A**) Hits number of the transcriptome of *R. spinulosa* against the databases of TF of *X. tropicalis*, *H. sapiens*, *M. musculus*, *G. gallus*, *D. rerio*, *D. melanogaster*, and *C. elegans*. (**B**) Transcription factor families of *R. spinulosa*. The families with less than 10 genes were grouped in the “less represented” group for clarity purposes.

We first identified the TF set to determine which TFs are involved in temperature adaptation in *R. spinulosa*, finding 1027 TF genes. Adding the previously annotated 347 TFs by Pastenes et al.^8^, we obtained 1374 genes coding TFs in *R. spinulosa* (for details, see Supplementary FASTA format file). These TFs represent 2.03% of the total genes reported for this species. This result is similar to the number of *X. tropicalis* TF reported*,* which has 1235 TF genes^10^. From the BLASTx analysis, we found that *X. tropicalis* was the species with the most significant number of hits (n = 433), whereas *C. elegans* had the smallest number of hits (n = 5) (Fig. 1A).

After classifying by TF family, we found that the three most abundant TF families in *R. spinulosa* are the C2H2 zinc-finger (n = 347), homeodomain (n = 155), and basic helix-loop-helix (n = 84) (Fig. 1B). To better visualize the TF family bar plot, each TF family with a small number of members was grouped together in the “Less represented” category (Fig. 1B).

**Figure 2:**
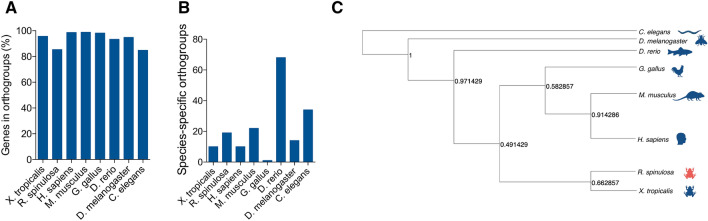
Ortholog transcription factors in *R. spinulosa*. (**A**) Percentage of TF of *R. spinulosa* in orthogroups with *X. tropicalis*, *H. sapiens*, *M. musculus*, *G. gallus*, *D. rerio*, *D. melanogaster*, and *C. elegans*. (**B**) Specific orthogroups number for each species. (**C**) Phylogenetic tree comparing the TF relationship of *R. spinulosa* with *X. tropicalis*, *H. sapiens*, *M. musculus*, *G. gallus*, *D. rerio*, *D. melanogaster*, and *C. elegans*.

The orthogroup analysis grouped at 94.3% of TFs in 1063 orthogroups (Fig. 2A). Moreover, the OrthoFinder assigned species-specific orthogroups for all the TFs, being *Danio rerio* the species with the most amounts of TFs (Fig. 2B). Additionally, the phylogenetic analysis with the TF sequences showed a closer relationship between *R. spinulosa* and *X. tropicalis* (bootstrap value: 0.66) than the other taxonomic groups, thus confirming the phylogenetic relationships (Fig. 2C).

### Abnormal expression of TFs in tadpoles from the El Tatio geothermal streams

**Figure 3:**
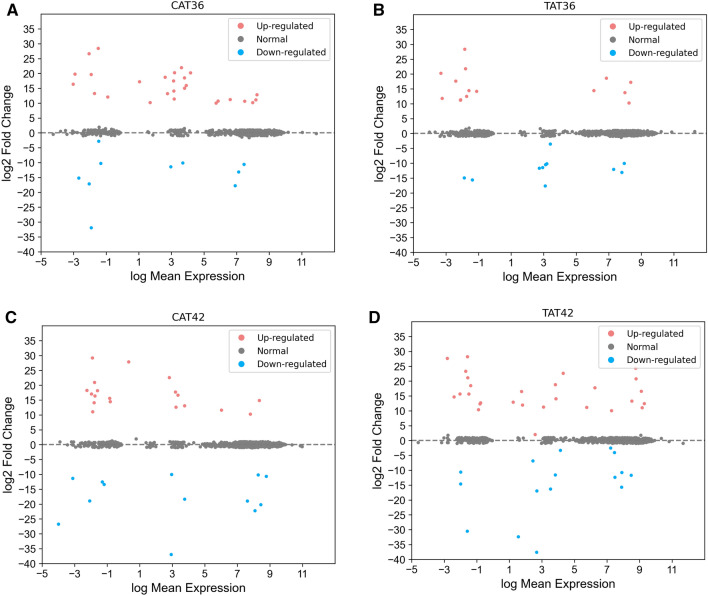
Up and down-regulated TFs at different temperatures and developmental stages. TFs log2 fold-change in the experimental conditions: (**A**) CAT36, Catarpe in Gosner 36; (**B**) TAT36, El Tatio in Gosner 36; (**C**) CAT42, Catarpe in Gosner 42; and (**D**) TAT42, El Tatio in Gosner 42.

Catarpe tadpoles at Gosner 36 exhibit 27 up-regulated and 10 down-regulated TFs (Fig. 3A). In contrast, for El Tatio tadpoles at the same developmental stage, we found only 14 up-regulated and 11 down-regulated TFs (Fig. 3B). Catarpe tadpoles at Gosner 42 exhibited 19 up-regulated and 13 down-regulated TFs (Fig. 3C). In the same developmental stage, El Tatio tadpoles presented 28 and 16 up- and down-regulated TFs, respectively (Fig. 3D).

### *Rhinella spinulosa* TFs in the prometamorphic and metamorphic stages

In the analysis of Catarpe tadpoles at Gosner 36, we discovered a significant up-regulation of TFs associated with cellular differentiation, central nervous system, lung, and vascular smooth muscle development (Table 1). Conversely, several TFs exhibited down-regulation in tadpoles of this population, which is associated with liver development, caudal body and spinal cord development, bone remodeling, and heart development (Table 1).

**Table 1:** Up-regulation of transcription factors during development.

Population	Gene	Function	Stage	Regulation	References
El Tatio	Neurogenin 3	Neuronal proliferation and differentiation	G36	Up	^37^
	T-cell leukemia homeobox protein 1	Neurogenesis and stem cell maintenance	G36	Up	^38^
	nkx-5	Brain development	G36	Up	^39^
	tgif2	Brain development	G36	Up	^40^
	foxc1	Vascular development	G36	Up	^22^
	trps1	Bone and cartilage differentiation	G36	Up	^41^
	glis2	Renal function	G36	Down	^42,43^
	hox-c11a	Femur, fibula, and kidney development	G36	Down	^44^
	znf268	Liver development	G42	Up	^45^
	dlx6	Craniofacial skeleton development	G42	Up	^46^
	fosl2	Bone development	G42	Up	^47^
	xsox-11	Neurogenesis	G42	Up	^48^
	six1	Muscle and organ development	G42	Up	^49^
	six2	Kidney development	G42	Up	^50^
	nr2e3	Photoreceptor differentiation and survival	G42	Up	^51^
	rora	Photoreceptor differentiation and survival	G42	Up	^52^
	gfi1	Lung development	G42	Down	^53^
	foxe1	Thyroid development	G42	Down	^54,55^
	gsx1	Brain development	G42	Down	^56^
Catarpe	nkx-2.4	Cellular diferentiation	G36	Up	^57^
	Myelin transcription factor	Cellular diferentiation	G36	Up	^58^
	rest co-repressor 3	Neurogenesis	G36	Up	^59^
	sox10	Glial cell differentiation	G36	Up	^60^
	hoxc5	Lung development	G36	Up	^61^
	meox2	Vascular smooth muscle	G36	Up	^62^
	znf268	Liver development	G36	Down	^45^
	cdx4	Caudal body and spinal cord development	G36	Down	^63^
	nkx-2.8	Bone remodeling and heart development	G36	Down	^64^
	hoxa5	Skeletal development and organogenesis	G42	Up	^65^
	tet2	Organ development	G42	Up	^66,67^
	hivep3	T-cell function	G42	Up	^68^
	vsx1	Retina development	G42	Down	^69^
	pax6	Eye development	G42	Down	^70^
	pou4f3	Inner-ear hairy cell development	G42	Down	^71^
	ptf1a	Pancreas development	G42	Down	^72^
	foxl1	Gut development	G42	Down	^73^

Shifting focus to the El Tatio tadpoles at Gosner 36, we found TFs involved in neuronal proliferation and differentiation, neurogenesis and neural stem cell maintenance, brain development, vascular development in the telencephalon, and bone and cartilage differentiation (Table 1). On the contrary, TFs linked to renal function and the development of the femur, fibula, and kidney showed down-regulation in these tadpoles.

Moving on to the analysis of Catarpe tadpoles at Gosner 42, we observed a striking up-regulation of TFs implicated in skeletal development and organogenesis, organ development, and T-cell function. Conversely, TFs integral to the retina and eye development, inner-ear hairy cell development, and pancreas and gut development, showed decreased expression levels (Table 1).

In the El Tatio tadpoles at Gosner 42, up-regulation was prominent TFs that guide bone, liver, muscle, and kidney development, neurogenesis, photoreceptor differentiation, and survival. On the other hand, TFs related to caudal body, lung, thyroid, and brain development exhibited down-regulation (Table 1). This comprehensive analysis highlights the intricate regulation of transcription factors in these populations, shedding light on their roles in various developmental processes.

### *Rhinella spinulosa* TFs involved in thermal adaptation

In Catarpe tadpoles at the G36 stage, up-regulation of TFs is associated with chronic stress responses, apoptosis suppression, and heat-shock protein expression (Table 2). Conversely, the El Tatio tadpoles at the same developmental stage only up-regulated one TF associated with hsp90 and hsp70 expressions (Table 2).

**Table 2:** Up-regulated transcription factors related to stress and thermal change.

Population	Gene	Function	Stage	Regulation	References
El Tatio	foxc1	Regulate expression of HSP70 and HSP90	G36	Up	^22,23, 29^
	foxo1	Regulate heat-shock protein expression	G42	Up	^24^
Catarpe	zbtb7a	Reduce oxidative stress	G36	Up	^25^
	atf7	Apoptosis suppression	G36	Up	^26–28^
	foxo1	Regulate heat-shock protein expression	G36	Up	^24^
	androgen receptor	Interaction with HSP56, HSP70, and HSP90	G42	Up	^30–33^
	af4/fmr2	Regulation of gene expression induced by thermal shock	G42	Up	^34^

On the other hand, in Catarpe tadpoles at the G42 stage, an up-regulation of TFs related to hsp90, hsp70, and hsp56 (Table 2). In contrast, the El Tatio tadpoles at the same developmental stage up-regulate a TF-regulating heat shock protein expression (Table 2).

## Discussion

Environmental stressors, particularly temperature fluctuations, significantly challenge the organism’s survival and thriving. TFs emerge as pivotal players in deciphering and transducing these stress signals, enabling organisms to adapt and persist. In this context, the present study unravels TFs-mediated stress responses in two distinct *Rhinella spinulosa* populations.

Our analysis revealed 1374 TF genes to *R. spinulosa*, encompassing diverse families, being C2H2 zinc-finger, homeodomain, and basic helix-loop-helix, the most abundant families. The observed prevalence of C2H2 zinc-finger, homeodomain, and basic helix-loop-helix TFs families in *R. spinulosa* is not exclusive to this anuran species. Our results align with findings of what is known to the human TFs, suggesting a degree of evolutionary conservation in the TFs family abundances across distant taxa^11^. The prominence of these families reflects their fundamental roles in orchestrating gene expression across a spectrum of biological processes, including from development to stress responses^12–15^.

Amphibians have a tolerable temperature range that allows their survival, which is restrained by the lower and upper critical thermal limits^16^. During ontogeny, the thermal tolerance of amphibians changes according to morphological and physiological reorganizations^17–19^. So, the thermoregulatory behavior allows them to adjust their body temperature and overcome temperature changes^20^. However, lower water temperature fluctuations restrict this behavior in amphibian larvae stages^21^. In the case of *R. spinulosa*, the Catarpe population experiences a habitat with frequent thermal changes, which contrasts with the constant temperature of water for the El Tatio population^9^. The adaptation of *R. spinulosa* to the thermal condition of El Tatio prevents them from adapting to a colder temperature^9^. Our results revealed that the Catarpe population up-regulated six TFs related to the regulation of heat-shock proteins (HSP) expression and apoptosis suppression. Conversely, El Tatio population only up-regulated foxc1 TF, which is associated with the regulation of the expression of HSPs^22^. Therefore, these results suggest that the inability of the El Tatio population to overcome thermal stress is partly explained by the lack of response at the TF level, indicating a local thermal adaptation.

In Catarpe population, the prometamorphic tadpoles up-regulated foxo1, zbtb7a, and atf7 transcription factors. Foxo1 TF has been associated with a protective role under stress by inducing the expression of antioxidant enzymes, thus protecting the negative effects of reactive oxygen species^23,24^. The zbtb7a TF protects cells against oxidative stress-induced apoptosis^25^. Atf7 is a TF that is phosphorylated under stress conditions^26–28^. Conversely, the prometamorphic tadpoles from the El Tatio only up-regulated foxc1 TF, which is crucial for oxidative stress protection and cell viability^29^. In this sense, Mendez et al.^9^ showed that the El Tatio tadpoles couldn’t adapt to the thermal change from 25 to 20 °C. Like this, up-regulating only one TF related to stress is insufficient to overcome the thermal stress, contrasting with the adaptive TF response shown by the Catarpe tadpoles.

On the other hand, Catarpe metamorphic tadpoles up-regulated the androgen receptor (AR) and af4/fmr2 transcription factor. AR, a nuclear receptor transcription factor, forms a complex with the molecular chaperones and co-chaperones hsp56, hsp70, and hsp90^30^. These chaperones play a key role in maintaining appropriate protein folding under thermal stress^31^. Furthermore, AR regulates cell survival and apoptosis by regulating the p53 pathway and reducing the expression of the apoptotic protein caspase-2^32,33^. Af4/fmr2 TF has been involved in the Ras/MAPK and PI3K/PKB pathways controlling cellular growth and identity^34^. In contrast, the metamorphic tadpoles from the El Tatio up-regulated foxo1, which involves heat-shock proteins expression. This population up-regulated only one TF, making them vulnerable to thermal environmental changes.

## Conclusions

TFs govern all aspects of cellular function, making them key players in adaptation processes. Our study underscores the importance of TF-mediated responses in enabling organisms to adapt to environmental stressors, particularly temperature fluctuations. It highlights the significance of local adaptation in shaping these responses. The comparison of two temperature treatments revealed that the Catarpe tadpoles up-regulated three TFs in the prometamorphic stage and two in the metamorphic stage. In contrast, the El Tatio tadpoles increased the expression of only one TF in each studied stage. This difference in the expression of TFs underlies the incapacity of the El Tatio tadpoles to overcome thermal changes. Further exploration of TF dynamics in response to environmental stressors will enhance our understanding of adaptive mechanisms in amphibian native populations and aid in conservation efforts amidst changing climates.

## Material and methods

To screen DNA-binding and TF proteins associated with the thermal local adaptation, we employed the consensus transcriptome and transcripts data (https://www.ncbi.nlm.nih.gov/sra/?term=rhinella+spinulosa) obtained from the common garden experiment and RNA-Seq assay carried out by Pastenes et al.^8^. Briefly, these authors used two larvae groups from the El Tatio at the Gosner 25 stage, which were independently exposed to 20 °C and 25 °C until reaching the Gosner 36 (G36) and Gosner 42 (G42) developmental stages. This same procedure was replicated for two larvae groups from Catarpe. For more detailed information about the common garden experimental design, RNA-Seq assay, transcriptome assembly, and *Rhinella spinulosa* Transcriptome Database, refer to Pastenes et al.^8^.

### Identification of DNA-binding and TF proteins from the annotated functional genes

We used the complete set of transcript gene models from the *Rhinella spinulosa* Transcriptome Database^8^ to identify the TFs previously annotated. First, we constructed a database with the gene, transcript ID, and annotated gene names. From the total assembled genes (87,844), we got the coding sequences (CDS), reducing to a total of 67,848 genes. Then, we filter the genes according to the “transcription” and “DNA-binding” gene names, getting 1068 genes. Finally, a manual gene cure was performed to consider only those corresponding to transcription factors or DNA-binding proteins, comprising a list of 347 TFs.

### Identification of TF proteins from the AnimalTFDB database

To identify potential TF genes in *R. spinulosa,* we use the remaining CDS, corresponding to a list of 67,501 genes. Additionally, we downloaded the complete set of *Homo sapiens* (1665), *Mus musculus* (1636), *Gallus gallus* (1134), *Xenopus tropicalis* (1236), *Danio rerio* (2547), *Drosophila melanogaster* (651), and *Caenorhabditis elegans* (748) TF proteins from the AnimalTFDB 3.0 database^35^, because it is a comprehensive and well-known database for the prediction and classification of transcription factors genes. The downloaded sequences correspond to homolog and ortholog transcripts of *R. spinulosa*. Next, we used BLASTx (https://blast.ncbi.nlm.nih.gov/Blast.cgi) to compare the 67,501 genes against the downloaded TF proteins, using the following criteria: E-value below 1e-10, 30% of sequence coverage, and 30% of sequence identity. Furthermore, when a gene from *R. spinulosa* exhibited alignment with multiple transcription factors (TFs) from several species, we selected the genes with the lowest E-value. Also, when a TF sourced from a species cataloged in the AnimalTFDB database displayed alignment with several genes of *R. spinulosa*, we prioritized the hit with the lowest E-value from the obtained results.

### Identification of the TF gene names

To assign gene names to the identified TFs, we performed a systematic analysis based on close-species protein match to support name identification. First, we used the BLASTx outputs described above, generating the closest known protein match from *Homo sapiens*, *Mus musculus*, *Gallus gallus*, *Xenopus tropicalis*, *Danio rerio*, *Drosophila melanogaster*, and *Caenorhabditis elegans* species for each gene sequence. Next, TFs were classified based on the structure of their DNA-binding domains into 22 family groups. The TF families containing 1 to 9 members were classified as “Less represented,” and TFs without a classification were represented as “Others.”

### Homology of TFs

To analyze the evolutionary history of *R. spinulosa* TFs, we first translated the transcripts into protein sequences according to the CDS information obtained from the *Rhinella spinulosa* Transcriptome Database^8^, namely in the first frame of forward or reverse strand as appropriate, using the ExPASy Translate tool (https://web.expasy.org/translate/). Subsequently, we used OrthoFinder v2.5.5^36^ (https://github.com/davidemms/OrthoFinder) with default settings to identify shared gene families among *R. spinulosa* and seven other species, including primate (*Homo sapiens*), rodent (*Mus musculus*), bird (*Gallus gallus)*, fish (*Danio rerio*), insect (*Drosophila melanogaster*), nematode (*Caenorhabditis elegans*) and another amphibian (*Xenopus tropicalis*), to infer a phylogenetic tree using their TF protein sequences. The Orthofinder software employs the maximum likelihood (RAxML) method. The numbers at the nodes in the consensus tree represent the bootstrap analysis values (100 replicates by default).

### Identification of up and down-regulated TFs

We assessed whether the thermal switch (25 °C to 20 °C) in *R. spinulosa* from Catarpe and El Tatio induces changes in TFs expression at Gosner 36 and Gosner 42 stages, we compared the log2 FPKM of each TFs family in different temperature conditions (for review raw data of FPKM values see Table S1).

To identify the TFs that were up and down-regulated by the temperature switch (25 °C vs. 20 °C), we use the previously calculated log2 fold-change by Pastenes et al*.*^8^ (i.e., log2[FPKM 25 °C/FPKM 20 °C]). We considered a log2 fold-change bigger than 2 as up-regulated and less than -2 as down-regulated.

### Plots

All the data was plotted using GraphPad Prism v10 and Python3 libraries (https://docs.python.org/3/library/index.html).

### Supplementary Information

Supplementary Information 1.

Supplementary Information 2.

Supplementary Table S1.

## Data Availability

Data are provided within the manuscript or in supplementary information files.
