# Supplementary material for: Transcription factor roles in the local adaptation to temperature in the Andean Spiny Toad Rhinella spinulosa
Source: Sci Rep. 2024 Jul 2;14:15158. doi: 10.1038/s41598-024-66127-5 (PMC11220030; doi:10.1038/s41598-024-66127-5)
Supplement: Supplementary file 2 — Supplementary Information 2. [file 41598_2024_66127_MOESM2_ESM.docx]

**Manuscript Title:** Transcription factor roles in the local adaptation to temperature in the Andean Spiny Toad *Rhinella spinulosa*

**Supplementary Data**

**Supplementary material legends**

**Table S1:** FPKM values of the transcription factors in Catarpe and El Tatio tadpoles according to thermal treatment (25 ºC or 20 ºC). TFs are sorted according to the gene family (TF family). Genetic information associated with the *Rhinella spinulosa* transcriptome database (RSV name and gene identifier) is included. Abbreviations: CAT20G36, Catarpe at 20 ºC in Gosner 36; CAT20G42, Catarpe at 20 ºC in Gosner 42; CAT25G36, Catarpe at 25 ºC in Gosner 36; CAT25G42, Catarpe at 25 ºC in Gosner 42; TAT20G36, El Tatio at 20 ºC in Gosner 36; TAT20G42, El Tatio at 20 ºC in Gosner 42; TAT25G36, El Tatio at 25 ºC in Gosner 36; and TAT25G42, El Tatio at 25 ºC in Gosner 42. (Submitted as Excel file)

**FASTA format file:** Protein sequences of the 1,374 transcription factors. (Submitted as plain text file)
